# AI-driven multi-agent collaborations for accelerating catalyst design

**DOI:** 10.1093/nsr/nwag067

**Published:** 2026-01-31

**Authors:** Hao Li

**Affiliations:** Advanced Institute for Materials Research (WPI-AIMR), Tohoku University, Japan

The electrochemical nitrogen reduction reaction (eNRR) offers a sustainable pathway for ammonia synthesis under ambient conditions, representing a promising alternative to the energy-intensive Haber–Bosch process [1]. However, the development of efficient electrocatalysts is hindered by the sluggish kinetics of N≡N bond cleavage and the competing hydrogen evolution reaction. While data-driven approaches and machine learning (ML) have accelerated the screening of catalysts, extracting reliable structure–activity relationships (SARs) from the vast and heterogeneous literature remains a formidable challenge. Large language models (LLMs) have recently demonstrated remarkable capabilities in scientific text mining, yet they often suffer from ‘hallucinations’ and a lack of deep domain reasoning when applied to specialized tasks like catalyst design [2].

**Figure 1.**
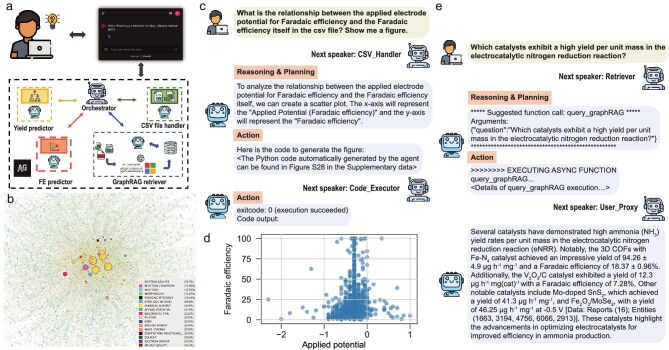
A comprehensive illustration of eNRRCrew. (a) Schematic image of eNRRCrew. (b) The LLM-generated knowledge graph of the eNRR corpus was built using the GPT-4o-mini. The size of each node represents the number of edges associated with it, while the color indicates the category to which the node belongs. (c) Dialogue showing the eNRRCrew providing Python code to analyze the relationship between applied electrode potential and Faradaic efficiency. (d) The scatter plot generated from the eNRRCrew automatically illustrates the relationship between applied potential and Faradaic efficiency. (e) Dialogue showing the eNRRCrew providing information regarding which catalysts exhibit a high eNRR yield per unit mass. Reproduced from Hu *et al.* [3].

Recently, a research team led by Prof. Xu Zhang from Zhengzhou University and Prof. Zhen Zhou from Nankai University developed eNRRCrew, a novel multi-agent collaboration framework that integrates LLMs, knowledge graphs and ML to automate the discovery of eNRR catalysts (Fig. 1) [3]. This work, published in *National Science Review*, represents a paradigm shift from isolated ML tasks to a holistic, agent-based research workflow.

The core innovation of eNRRCrew lies in its multi-agent architecture, which mimics a human research team. By deploying five specialized agents—an orchestrator for task delegation, yield/Faradaic efficiency (FE) predictors for performance forecasting, a GraphRAG retriever for evidence-based knowledge retrieval, and a CSV handler for data operations—the framework achieves seamless integration of literature mining, data analysis and predictive modeling. Analyzing over 2300 papers, the team constructed a comprehensive eNRR database. Through interpretable ML models (Random Forest), they identified critical descriptors that affect catalytic performance, such as the space group number and elemental electronegativity difference, providing physical insights often missed by ‘black-box’ models. Unlike traditional static databases, eNRRCrew enables natural language interaction. Researchers can ‘chat’ with the system to request novel catalyst recommendations. The system not only retrieves existing data but also proposes original catalyst candidates—such as an Mo–W dimer on Ti_2_NO_2_ MXene—and validates their thermodynamic stability via molecular dynamics simulations. Encouragingly, a recommended MoFeNC catalyst has recently been experimentally validated, confirming its promising activity [4]. This capability to generate and verify hypotheses autonomously is a significant leap forward.

In summary, Zhang and Zhou’s work demonstrates that LLM-based multi-agent systems can transcend simple information retrieval to become active participants in scientific discovery. By addressing the specific challenges of eNRR (low selectivity and yield) through automated SAR analysis, eNRRCrew offers a scalable template. This methodology can be readily extended to other complex domains, such as the oxygen evolution reaction (OER) or material design, heralding a new era of ‘AI-Crew’-driven materials science [5].
